# Late, not early mismatch responses to changes in frequency are reduced or deviant in children with dyslexia: an event-related potential study

**DOI:** 10.1186/1866-1955-6-21

**Published:** 2014-07-25

**Authors:** Lorna F Halliday, Johanna G Barry, Mervyn J Hardiman, Dorothy VM Bishop

**Affiliations:** 1 Division of Psychology and Language Sciences, University College London, 2 Wakefield Street, London WC1N 1PF, UK; 2 MRC Institute of Hearing Research Nottingham Clinical Section, Eye, Ear Nose & Throat Centre, Queens Medical Centre, Nottingham NG7 2UH, UK; 3 Department of Experimental Psychology, University of Oxford, South Parks Road, Oxford OX1 3UD, UK

**Keywords:** Dyslexia, Mismatch negativity, MMN, Late discriminative negativity, LDN, Phase locking, Inter-trial coherence, Event-related spectral perturbation, Event-related desynchronization, Frequency discrimination

## Abstract

**Background:**

Developmental disorders of oral and written language have been linked to deficits in the processing of auditory information. However, findings have been inconsistent, both for behavioural and electrophysiological measures.

**Methods:**

In this study, we examined event-related potentials (ERPs) in 20 6- to 14-year-old children with developmental dyslexia and 20 age-matched controls, divided into younger (6–11 years, *n* = 10) and older (11–14 years, *n* = 10) age bands. We focused on early (mismatch negativity; MMN) and late (late discriminative negativity; LDN) conventional mismatch responses and associated measures derived from time-frequency analysis (inter-trial coherence and event-related spectral perturbation). Responses were elicited using an auditory oddball task, whereby a stream of 1000-Hz standards was interspersed with rare large (1,200 Hz) and small (1,030 Hz) frequency deviants.

**Results:**

Conventional analyses revealed no significant differences between groups in the size of the MMN to either large or small frequency deviants. However, the younger age band of children with dyslexia showed an enhanced inter-trial coherence in the theta frequency band over the time window corresponding to the MMN to small deviants. By contrast, these same children showed a reduced-amplitude LDN for the small deviants relative to their age-matched controls, whilst the older children with dyslexia showed a shorter and less intense period of event-related desynchronization over this time window.

**Conclusions:**

Initial detection and discrimination of auditory frequency change appears normal or even enhanced in children with dyslexia. Rather, deficits in late-stage auditory processing appear to be a feature of this population.

## Background

Developmental dyslexia (hereafter ‘dyslexia’) is typically diagnosed where a child has difficulty learning to read and spell, despite having no known physical, intellectual, neurological, emotional, educational, or socio-economic problems which might account for these difficulties [1]. Researchers largely agree on those factors that would preclude a diagnosis of dyslexia, yet the causal factors that lead to the disorder are still a matter of debate. There are now several influential theories which state that dyslexia, at least in a subset of individuals, arises from low-level impairments in the perception and processing of auditory information (e.g. [2-5]). These theories propose a variety of different auditory processing impairments as underlying dyslexia, from deficits in ‘rapid’ auditory temporal processing [5], to frequency discrimination [6], and the detection of rate of change of amplitude at the onset (rise time) and/or during the speech envelope [2,3]. Nonetheless, they are united in the premise that these impairments lead to difficulties in analysing the incoming speech stream and subsequent problems with phonological processing (i.e. in discriminating, categorizing, and manipulating speech sounds). In turn, these difficulties with phonological processing are thought to lead to problems in learning to read in the case of dyslexia (cf. [7]). A subset of these theories also accounts for the oral language problems of those with specific language impairment (SLI) (e.g. [3,5]).

There is now a large number of studies showing that both children and adults with dyslexia tend to perform more poorly than matched peers on behavioural measures of auditory processing (for review, see [8]). These measures include, but are not limited to, those of auditory frequency discrimination (i.e. the ability to discriminate differences in pitch) [8]. However, not all studies have replicated findings of deficits in auditory processing in this population (for reviews, see [8,9]). One factor that may contribute to these mixed results is the nature of the psychophysical tasks used to assess auditory processing. Behavioural evidence can be difficult to interpret, as elevated thresholds on such tasks can arise due to poor (selective or sustained) attention, memory, or motivation, even when stimuli can be accurately discriminated [10-12]. These confounds are characteristic of children, but particularly of children with dyslexia, who frequently also experience deficits in attention and memory in addition to their reading difficulties [13-17]. Researchers have therefore turned to the auditory event-related potential (ERP), in the hope that it will provide a more objective measure of auditory processing in dyslexia.

The mismatch negativity (MMN) component of the ERP has rapidly become the method of choice for assessing auditory discrimination in dyslexia. The auditory MMN is typically elicited using an oddball paradigm in which a train of repeated standard auditory stimuli includes occasional deviant stimuli that differ from the standard in one or more acoustic dimensions (e.g. frequency, duration, or rise time; [18]). Responses to standards and deviants are averaged separately and then subtracted from each other. Typically, this method reveals the presence of an enhanced negative response to deviants, occurring approximately 100–250 ms after stimulus onset, with the latency and amplitude of the negativity increasing with the difference of the deviant from the standard tone [19,20]. Hence, it is often viewed as a discriminative response. Importantly, the MMN can be elicited passively, i.e. without the need for participants to perform a task or attend to the stimuli. Note that this paradigm has also been shown to elicit a component known as the late discriminative negativity (LDN) [21] (also ‘late MMN’ [22]), which is reflected as a prolonged period of negativity occurring around 300–550 ms post-stimulus onset [21]. Unlike the MMN, there is evidence that the LDN is larger for small rather than large deviants and may reflect additional processing of auditory stimuli that occurs when the salient features of the stimulus are difficult to discriminate [23].

Given that the MMN is an objective measure of auditory discrimination, it is surprising that studies assessing the auditory MMN in dyslexia often yield less consistent findings than behavioural studies [8]. For instance, studies assessing responses to frequency deviants have demonstrated an MMN in dyslexic groups that is reduced [24-26], enhanced [27], or not significantly different in amplitude from that of matched controls [28-32]. Other studies have found that whether or not a dyslexic group showed differences in the MMN depended on the particular electrode(s) chosen for analysis, stimuli used, or participant characteristics [33-38]; (see also [39] for review). In a review of the literature, Bishop [40] suggested that inconsistencies in the reported findings may reflect a combination of methodological differences between studies, including differences in statistical power, participant characteristics, stimuli, and presentation rate, as well as factors associated with the measurement of the MMN itself (e.g. the specific time window used to define the MMN—and whether or not it overlaps with the LDN time window). These factors, combined with the poor reliability of the MMN at the individual level (e.g. [41,42]), have led some researchers to question whether the MMN is truly the gold standard measure of auditory discrimination ability that it was initially hailed to be [43].

In addition to traditional MMN techniques which involve averaging ERPs, further information can be obtained by analysis of the component waveforms that underlie the MMN. The background electroencephalogram (EEG) comprises an ensemble of cortical oscillations across a range of different frequencies (from delta (0–4 Hz) through theta (4–7 Hz), alpha (8–12 Hz), beta (12–30), and gamma (30–100 Hz)). One explanation of the auditory sensory ERP is that following the presentation of a sound, cortical oscillatory activity is synchronized in phase with the incoming auditory signal (‘phase resetting’ [44]). Magnetoencephalography (MEG) and EEG studies have revealed an increase in phase locking of oscillatory activity to deviant auditory stimuli during the MMN time window, but confined to the theta range only [23,45-47]. In contrast, the LDN has been linked to an event-related *desynchronization* of oscillations extending across the delta, theta, and alpha ranges [48]. These responses showed strong developmental effects, with cortical synchronization in the theta band corresponding to the MMN increasing with age in typically developing children ranging from 7 years to adulthood [23]. In addition, synchronization in the theta band over the MMN time window was significantly correlated with behavioural thresholds on a measure of frequency discrimination [23] (c.f. [48]). Of particular relevance to the current study, Bishop et al. [48] found that children with SLI failed to show the expected event-related desynchronization during the LDN time window that was seen in typically developing controls. Moreover, the drop in event-related spectral power associated with the LDN to syllables was correlated with performance on a measure of nonword repetition (a measure of phonological processing and potential gold standard test for language impairment [49]), indicating that it was those children who were poor at nonword repetition that failed to show desynchronization.

The relationship between SLI and dyslexia is not clear cut (e.g. [50]). However, as many as 50% of children with SLI have reading difficulties [51], and both children with SLI (e.g. [52]) and children with dyslexia (e.g. [53]) are poor at nonword repetition. Given that dyslexia is also associated with difficulties with phonological processing (see [50] for review), we would expect to see similar patterns of brain activity in children who have experienced difficulties in learning to read.

In the current study, we compared mismatch responses to changes in auditory frequency in a group of children with dyslexia and a group of typically developing controls who were matched in age. We used a subset of the stimuli and an identical paradigm to that of Bishop and colleagues [23,48], in which a repeated standard stimulus (a sinusoid of a set frequency) was interrupted by two rarer deviant sinusoids of different frequencies. We compared the responses of the two groups in both the MMN and LDN time windows, using both conventional techniques, and time-frequency analyses of spectral composition and phase locking. We asked: (1) Are children with dyslexia different from typically developing controls in their mismatch responses to frequency deviants during the MMN or LDN time windows when assessed using conventional analysis techniques? (2) Are children with dyslexia different from typically developing children in the spectral composition or in the extent of phase locking of the MMN or LDN? (3) Are any measures of the MMN or LDN related to behavioural measures of frequency discrimination and oral or written language in this group? Following Bishop et al. [48], we predicted that like children with SLI, children with dyslexia would show an MMN response that was no different from that of typically developing controls, but that their LDN would be reduced in amplitude, and that this would be particularly pronounced for the small deviants. Further, we predicted that children with dyslexia would show normal phase locking but reduced desynchronization of activity relative to controls. Finally, based on past research, it was difficult to predict whether any of the EEG measures would correlate with frequency discrimination or language/literacy [23,48], but we hypothesised that any significant correlations would be restricted to between frequency discrimination and phase locking to small deviants, and between event-related desynchronization and nonword repetition.

## Methods

### Participants

Twenty 6–14-year-old children with developmental dyslexia (DYS group) were recruited from specialist, independent, and mainstream schools in the South East of England. All had received a diagnosis of dyslexia from an educational psychologist and scored more than one SD below the mean on a standardized measure of word or nonword reading (Test of Word Reading Efficiency [54], *n* = 8), nonword repetition (NEPSY [55], *n* = 3), or both (*n* = 9) (see below for descriptions of these standardized assessments). Typically developing control children (TD group, *n* = 20) were a subset of those studied by Bishop et al. [23] selected to be matched on age to the children with dyslexia. All children spoke English as their first language and were not fluent speakers of another language. All had nonverbal abilities of ≥80 on the Matrices Reasoning subtest of the Wechsler Abbreviated Scale of Intelligence (WASI [56]; see ‘Nonverbal ability’ section) and had pure tone audiometric thresholds within normal limits (<30 dB HL at 1, 2, and 4 kHz, allowing for a non-sound-attenuated testing environment; [57]). The participants were divided into older and younger subgroups (‘older’ and ‘younger’) on the basis of a median split (10.5 years). Table 1 shows the characteristics of the four subgroups.

**Table 1:** Mean (SD) age and scores on the questionnaire, psychometric and psychophysical test batteries

	**Group**				**Statistics ( *p *; (*n***^**2**^**))**		
	**TD**		**DYS**				
	**Younger**	**Older**	**Younger**	**Older**	**Group**	**Age band**	**Group × age band**
Number of males and females	5 males, 5 females	5 males, 5 females	9 males, 1 female	6 males, 4 females			
Age (years)	8.96 (1.08)	11.93 (1.19)	9.06 (0.99)	12.32 (0.74)	0.461 (0.02)	*<0.001 (0.72)*	0.659 (0.01)
CCC-2 GCC^ab^	91.20 (18.16)	84.10 (12.84)	57.30 (23.80)	59.11 (22.44)	*<0.001 (0.38)*	0.662 (0.01)	0.485 (0.014)
WASI matrices^c^	108.50 (12.48)	102.90 (9.01)	98.20 (15.70)	98.10 (10.29)	0.054 (0.10)	0.460 (0.02)	0.478 (0.01)
TOWRE sight words^c^	104.60 (11.06)	97.40 (11.66)	87.40 (12.23)	80.90 (10.31)	*<0.001 (0.36)*	0.06 (0.092)	0.923 (0.00)
TOWRE phonetic decoding^c^	108.70 (8.60)	107.90 (12.74)	82.60 (7.29)	71.15 (8.28)	*<0.001 (0.76)*	*0.05 (0.10)*	0.080 (0.08)
NEPSY nonword repetition^d^	10.80 (2.20)	11.60 (2.67)	6.90 (2.02)	6.60 (2.27)	*<0.001 (0.50)*	0.732 (0.00)	0.455 (0.016)
FD threshold (log 10 Hz)^a^	1.87 (0.32)	1.11 (0.54)	1.49 (0.54)	1.11 (0.21)	NA	See text	NA
FD threshold (Hz)^a^	93.83 (61.46)	28.37 (43.86)	54.00 (53.90)	14.29 (6.65)	NA	NA	NA

*GCC* General Communication Composite of the Children's Communication Checklist (CCC-2) (Bishop [58]), *WASI* Wechsler Abbreviated Scale of Intelligence (Wechsler [56]), *TOWRE* Test of Word Reading Efficiency (Torgesen et al. [54]), NEPSY (Korkman et al. [55]), *FD* frequency discrimination. See text for details. ^a^Missing data: one case for CCC-2, two for FD. ^b^Normative mean = 80. ^c^Scaled with mean 100, SD 15. ^d^Scaled with mean 10, SD 3. The italicised values indicate those comparisons that are significant at *p* < 0.05.

The study was approved by the Oxford Psychiatric Research Ethics Committee. The parents of all participants gave written informed consent, and the children gave verbal assent after the study was explained in age-appropriate language.

### Psychometric assessments

#### ***Nonverbal ability***

Nonverbal ability was estimated using the Matrices Reasoning subtest of the WASI [56]. The subtest is composed of 35 items graded in difficulty and measures four types of nonverbal reasoning: pattern completion, classification, analogy, and reasoning. For each item, the participant selects one of five patterned segments to complete a large patterned matrix that contains a blank segment. Scores on the matrices reasoning test are expressed as scaled scores, with a mean of 100 and a standard deviation of 15.

#### ***Communication***

Communication abilities were assessed using the Children's Communication Checklist (CCC-2) [58], a parent and/or teacher checklist designed to obtain ratings of aspects of communication. Items are divided into ten scales, and the scores on the first eight of these scales (speech, syntax, semantics, coherence, inappropriate articulation, stereotyped language, use of context, and nonverbal communication) are used to derive a General Communication Composite (GCC), which can be used to identify children likely to have clinically significant communication problems. A score of less than 55 on the GCC has been shown to select the bottom 10% of children in respect of communication abilities [59].

#### ***Reading***

Reading accuracy and fluency were assessed using version A of the Test of Word Reading Efficiency (TOWRE [54]) which comprises the Sight Word Efficiency and the Phonemic Decoding Efficiency subtests. Each subtest consists of a list of eight practice items and a longer list of test items, graded in difficulty. The Sight Word Efficiency subtest contains 104 familiar regular words, and the Phonemic Decoding Efficiency subtest contains 63 orthographically legal pronounceable nonwords. After successfully reading the practice items, the participant's task is to read as many words/nonwords aloud as possible in 45 s. As a measure of speed as well as accuracy, the TOWRE is thought to be especially sensitive to reading problems in people who have had extensive remediation. The scores are expressed as standard scores with a mean of 100 and a standard deviation of 15.

#### ***Phonological processing***

Receptive and expressive phonological processing was assessed using the Repetition of Nonsense Words subtest from the neuropsychological assessment NEPSY [55]. This test consists of 13 nonword items, ranging from two to five syllables in length, which were presented via a computer at a comfortable listening level. The original items from the NEPSY were re-recorded using a digital DAT recorder by a female native speaker of British English, in a sound attenuated booth. The participant's task is to repeat each nonword out loud. The scores are expressed as scaled scores with a mean of 10 and a standard deviation of 3. The scores for participants aged 13 or over were calculated from the norms provided for the oldest age group (12 years and 6 months to 12 years and 11 months).

### Psychophysical assessment of frequency discrimination

#### ***Stimuli and equipment***

Stimuli for the frequency discrimination task varied for the DYS and TD groups. For both groups, to-be-discriminated stimuli were pure tones, gated on and off by a 10-ms raised cosine ramp. The standard stimulus was a 1,000-Hz sinusoid tone, and the deviant stimulus varied in frequency between 1,200 and 1,001 Hz. For the DYS group, the stimuli were 1,000 ms in duration. For the TD group, the stimuli were 100 ms in duration. For the DYS group, the stimuli were presented on a laptop using Sennheiser HD-580 headphones (Wedemark. Germany), monaurally to the right ear, at a peak intensity of 75 dB SPL. For the TD group, stimuli were presented binaurally via Sennheiser HD-25 headphones, at 85-dB sound pressure level (SPL). Differences between the two tasks arose as data from the DYS group was being collected as part of a separate study for the first author's DPhil thesis [60].

#### ***Procedure***

The stimuli were delivered via a computer game (the ‘Dino task’, created by Dorothy Bishop, University of Oxford, [11]) using a self-paced AXB three-interval two-alternative forced-choice procedure. On each trial, the participants were played three tones, which were represented on the screen by three cartoon dinosaurs. The middle dinosaur always corresponded to the standard tone, and the first and the third dinosaurs either played another standard tone or a deviant tone that was higher in frequency. The participants were asked to select the dinosaur (either the first or the third) that made the same sound as the middle dinosaur. Correct responses were marked by the appearance of reward tokens on the screen, which were collected at the side of the screen as a record of past success and by the presentation of a ‘happy’ sound (a rising frequency sweep). Incorrect responses were signalled by the absence of a reward token and by the presentation of an ‘unhappy’ sound (a falling ‘sigh’). Each tone was separated by 500 ms of silence. A more virulent PEST procedure was used to adjust the difference between the standard and the deviant tone to converge on a 75% correct response rate on the psychometric function [61]. The difference between the standard and the deviant tone was initially adjusted in large step sizes (24 Hz), which were halved at each reversal (change in direction of the adaptive track from decreasing to increasing or vice versa, owing to accuracy on a given trial/sequence of trials). Tracks had an upper limit of 80 trials or 8 reversals (whichever came first). In general, two tracks per participant were given. The main test was preceded by a series of practice trials, during which the experimenter demonstrated the correct response to the participant and gave verbal feedback and encouragement until it was clear that the task had been understood. Thresholds for each track were calculated as the mean difference (in Hz) between the standard and deviant tones from the fourth reversal, converted to log base 10 to normalize the data. For those participants who completed two tracks (DYS group *n* = 18; TD group *n* = 16), frequency discrimination thresholds were calculated as the mean of the two track thresholds.

### Electrophysiological assessment of mismatch negativity

#### ***Stimuli***

A subset of the stimuli described in detail by Bishop et al. [23] was used in this experiment. The stimuli consisted of a standard 1,000-Hz tone and deviants of 1,200 Hz (large deviant) and 1,030 Hz (small deviant). All stimuli were 175 ms in duration and were gated on and off by a 15-ms raised cosine ramp. The stimuli were synthesized in Matlab, at a sampling rate of 44,100 Hz. The resulting waveforms were presented via a custom-made program through a PC with an audiocard with a 16-bit digital-to-analog converter. The stimuli were presented monaurally to the right ear via Sennheiser HD-25-1 headphones, at an intensity of 86.5-dB SPL.

#### ***Procedure***

The participants were tested in a sound-attenuated electrically shielded booth. The stimuli were presented over two blocks, each of 333 trials. A standard oddball paradigm was used, in which standards were presented on 70% of trials, with each deviant occurring on 15% of trials. This gave a total of 466 standards and 100 of each deviant type. An additional block was run if the preceding block appeared to have had excessive artefacts (DYS group *n* = 9; TD group *n* = 1). The stimuli were presented in a quasi-random sequence designed to ensure that at least two standard stimuli followed every deviant. The stimuli were presented at a constant stimulus onset asynchrony (SOA) of 1 s. The participants were instructed to ignore the stimuli and either played on a handheld game console or watched a silent video on a small television located 1.3 m away during the EEG recording.

#### ***EEG recording and data analysis***

The EEG was recorded on a SynAmps or NuAmps Neuroscan system (Charlotte, NC, USA) using Ag/AgCl-sintered electrodes and a water-soluble gel. Early pilot studies indicated no difference in the results obtained from the two recording systems. An electrode cap was fitted to record from 28 sites, according to the International 10/20 system [62]: FC1, F7, FP1, FZ, FP2, F8, FC2, FT9, FC5, F3, FCZ, F4, FC6, FT10, T7, C3, CZ, C4, T8, CP5, P7, P3, PZ, P4, P8, CP6, M1, and M2. M1 or M2 was selected as a reference, and the ground electrode was positioned at AFZ. The electrooculogram (EOG) was recorded using electrodes positioned laterally approximately 1 cm from the outer canthus of each eye and from supraorbital and infraorbital electrodes on the left eye. The EEG was recorded continuously online and stored for off-line processing. The EEG data were digitized at 500 Hz and bandpass filtered (0.01–70 Hz for SynAmps; 0.1–70 Hz for NuAmps) and had a 50-Hz notch filter. Impedances were kept below 8 kΩ.

#### ***Offline analysis***

Data processing is described in detail by Bishop et al. [23]. This was done separately for each individual participant. Raw EEG data were down-sampled to 250 Hz and high pass filtered at 0.5 Hz to remove drift. Re-referencing was performed to average mastoids to remove any lateral bias and to increase signal-to-noise ratio [63]. The data were epoched into trials of 1,000-ms duration, including a 200-ms baseline. The data were baseline corrected, and trials with extreme amplitudes (±350 μV) were rejected to remove noisy sections of record whilst retaining blinks. Artefact reduction was performed using second-order blind identification (SOBI) independent component analysis [64], implemented in EEGlab software [65] to identify unwanted components such as blinks, which were mathematically subtracted from the data. Further artefact rejection was then applied with a cutoff of ±150 μV. Spatial principal components were analysed on a participant-by-participant basis, treating channel amplitudes at each time point in the averaged waveform (all trials, standard, and deviant) as a new set of observations. Weights from the first component were then used to create a new channel consisting of the weighted average of all channels, with polarity set to be consistent with electrode FZ. Difference waveforms were created for single-trial analysis. This involved categorizing four types of trial—large deviants, small deviants, dummy deviants (i.e. standards preceding deviants), and all other standards. This latter set was averaged to give a mean standard response, which was then subtracted from all the other trials (see [45] for an analogous approach). We thus had three types of difference waveform, one for each deviant and one dummy set formed by subtracting each standard-before-deviant from the average of other standards. Note that the dummy difference waveform method was recommended by Picton and Taylor [66]. By comparing mismatches seen on these ‘dummy’ trials, where standards and dummy deviants were identical, with genuine mismatch trials, we could estimate the validity and reliability of mismatch responses. The mismatch responses were then low-pass filtered using an IIR filter with a cutoff of 30 Hz to smooth the peaks, and trials with absolute amplitudes greater than 100 μV were removed before baseline correction was reapplied. Finally, spatial PCA was re-run on the average difference wave for each deviant type, and the spectral power of the first component was computed for the frequency range 5 to 20 Hz (i.e. including theta and alpha frequencies) using the spectopo function from the EEGlab software [65]. This conducts a fast Fourier transform across the whole epoch, i.e. it estimates power in different frequency bands but is not sensitive to time.

Conventional analysis of mean amplitudes for each type of difference waveform (large deviants, small deviants, and dummy deviants), using time windows of 100–250 ms for the MMN and 350–550 ms for the LDN, was performed. Significance of mismatch responses at the group level was tested using *t* tests at each time point, comparing the group average amplitudes with zero. The likelihood of obtaining spurious differences was estimated by considering the dummy difference waves.

Time-frequency analysis of single trials was performed for each type of difference waveform using the EEGlab software [65] to obtain two spectrally based measures. The method for frequency extraction was fast Fourier transform with Hanning window tapering, and pad ratio was set to 2. This gave 200 estimates with subwindow centres ranging from -135.9 to 735.9 ms and a window size of 128 ms. Two measures were obtained from the time-frequency analysis. The first index, intertrial coherence (ITC), corresponds to the extent to which individual trials had oscillations in phase at a given frequency at a given time point. By comparing ITC at baseline and at stimulus onset, we can measure the degree to which responses are temporally aligned (or phase locked) to the auditory stimulus. ITC is a unitless measure, which ranges from zero to one, with one indicating perfect alignment, and zero indicating random phase alignment. The second index is event-related spectral perturbation (ERSP), which estimates the amount of power in the difference wave at a given frequency and a given time point, regardless of phase. It is expressed as a ratio and is measured here in decibels. We focused our analyses on the ITC and ERSP in the theta range that corresponded to the periods associated with the MMN and LDN, respectively. This follows the evidence that the MMN is characterized by a burst of increased ITC [48], whereas the LDN has been associated with a decrease in ERSP over the same time range [23].

### Data analysis

Prior to data analysis, each of the data sets was checked for outliers. Any data point that had a Cook's distance score >4/*N* (0.4) was removed in order to prevent that point having undue influence on the results. This resulted in the removal of one data point from the EEG data (0.16% of the points overall). Owing to time constraints, one of the TD group failed to complete the FD task. For the EEG analysis, we aimed to follow the analyses of Bishop et al. [23] using a series of repeated measures ANCOVAs on (i) MMN mean amplitude, (ii) ITC, (iii) LDN mean amplitude, and (iv) ERSP, with DYS status (DYS versus TD) and age band (younger versus older) as the two between-subject factors, and deviant (small versus large) as the within-subjects factor. Because the difference in nonverbal ability scores between the DYS and TD groups just missed significance (see Table 1), standard scores on the Matrices Reasoning subtest of the WASI [56] were also entered as a covariate. For each of the analyses, all main effects and interactions were initially entered into the model, and any nonsignificant interaction terms and, where permissible, main effects, were iteratively removed, until all remaining interactions and/or main effects were significant. For each of the analyses, only the final models are presented. We also planned to run a series of correlations to assess the association between the four EEG measures and behavioural measure of (i) frequency discrimination and (ii) language and literacy. Because of known developmental effects, age was included as a covariate in all correlation analyses.

## Results

### Frequency discrimination thresholds

The mean frequency discrimination thresholds for the four subgroups are shown in Table 1. Because of the differences in stimuli between the two tasks, it was not possible to statistically compare frequency discrimination thresholds of the two groups, as lower frequency discrimination thresholds have been found to be associated with tones of longer duration [67-69]. Instead, a univariate ANOVA was run for the two groups separately. For the TD group, there was a significant effect of age band, *F*(1, 17) = 14.21, *p* = 0.002, with the older age band showing significantly lower (better) thresholds than the younger age band. For the DYS, group, in contrast, this main effect was marginally nonsignificant, *F*(1, 36) = 4.37, *p* = 0.051, with the two age bands showing similar frequency discrimination thresholds. Note that the absolute values for the DYS group were similar to—albeit lower than—those obtained in the TD group using shorter stimuli (see Table 1).

### Electrophysiological responses to standards and deviants

The total number of deviant trials available for grand averaging differed significantly by DYS status, *F*(1, 42) = 16.91, *p* < 0.001, with the DYS group having significantly more than the TD group (DYS group 207.60, SD = 34.52; TD group 175.60, SD = 18.51). However, effects due to noisier grand averages are unlikely to be a factor in observations since all children had at least 60 deviant trials per condition that could be grand averaged, and the number of artefact-free trials did not correlate significantly with any of the ERP components examined here (see Additional file 1: Table S5).

**Figure 1:**
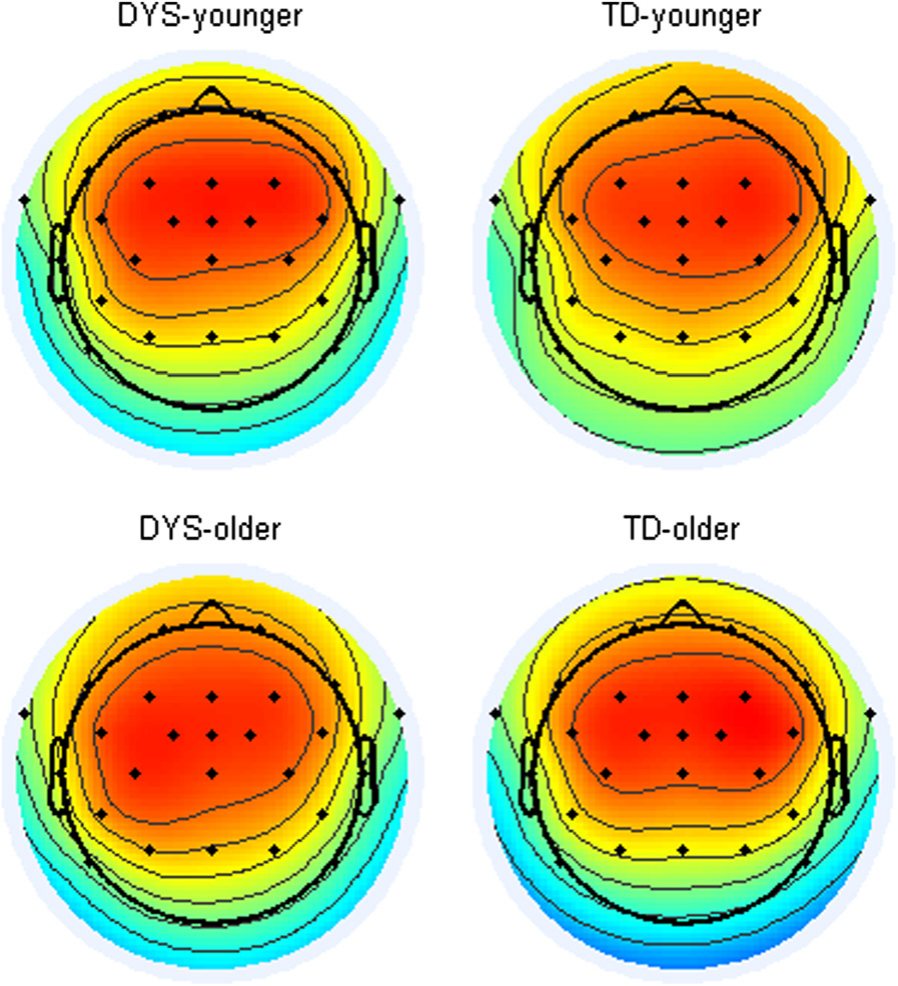
**Topographic headplots showing averaged weightings of electrodes on the first principal component.** Subplots divided according to DYS status and age band; arbitrary scaling from negative (blue) to positive (red).

We followed the procedure of Bishop et al. [23,48] and used the first spatial principal component to represent the auditory ERP. The mean weights from each channel contributing to the principal component for each subgroup are shown in headplots in Figure 1. Consistent with Bishop et al. [23,48], these indicated a fronto-central distribution that was similar between groups and across age groups, although slightly more left-lateralized in the dyslexic group, and particularly the older DYS subgroup.

**Figure 2:**
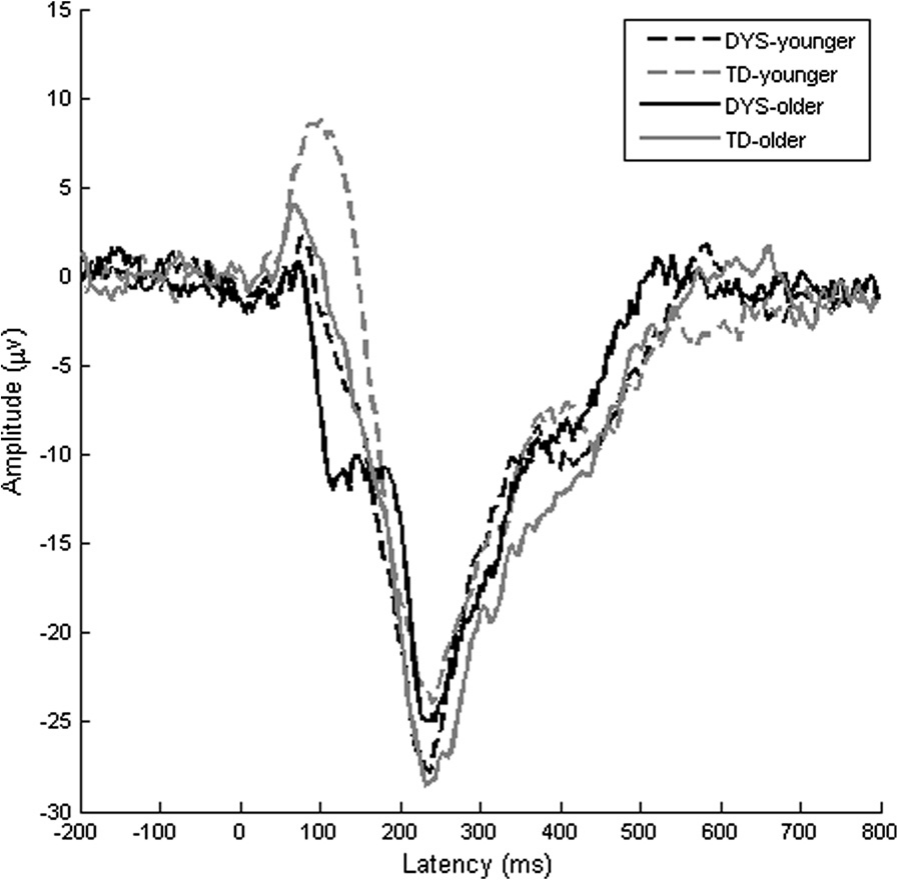
**Average amplitude at principal component.** Subplots divided according to DYS status and age band. Note that the average amplitude generated by principal components analysis is substantially larger than for the conventional N1-P2 response.

Responses to standards and deviants based on the spatial principal component are shown in Figure 2. Surprisingly, the DYS subgroups appeared to show a more mature pattern than the TD subgroups, with the DYS younger subgroup exhibiting a smaller P1 than their typically developing peers, and the DYS older subgroup showing what appeared to be a developing N1 component at around 100–150 ms. The TD younger subgroup, in contrast, showed a large P1 followed by a prolonged N2 component and a notable absence of the N1, whilst the TD older subgroup showed a smaller P1 but still no signs of the N1. These responses are consistent with previous literature for children of these ages at this inter-stimulus interval (e.g. [40]).

**Figure 3:**
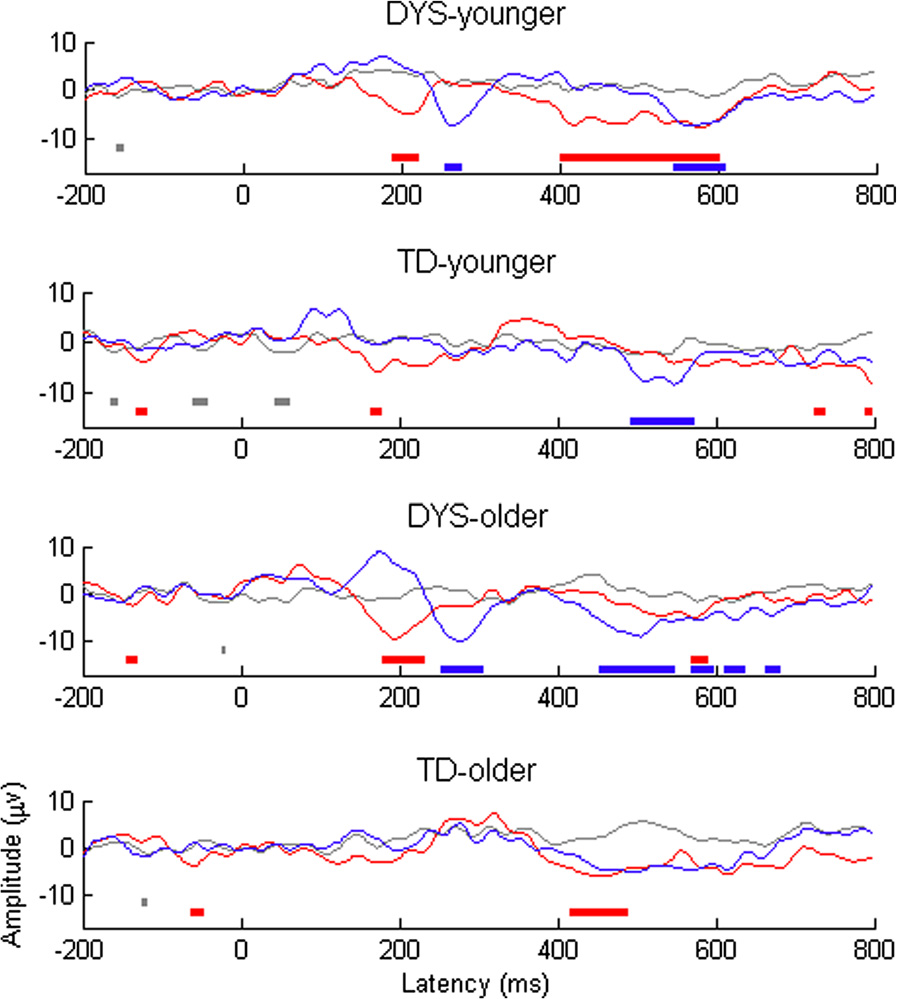
**Average mismatch responses from first principal component by DYS status and age band.** Dummy difference files are shown in grey, large deviants in red, and small deviants in blue. Regions where *t* test value is < -1.96 are shown as bars of same colour below the plot.

The difference waveforms for each subgroup are shown in Figure 3. One-sample *t* tests were conducted at each time point to identify regions where the averaged difference waveform for all participants fell significantly below zero. *T* values < -1.96 are shown below the waveforms in Figure 3. In order to give an indication of how much amplitude is to be expected by chance, dummy waveforms were created by subtracting the average for standards from each standard-before-deviant. These show the likelihood of obtaining spurious differences as a result of conducting multiple comparisons across time points that are not independent of one another. As illustrated in Figure 3, both the younger and the older DYS groups showed a short period of enhanced negativity occurring around 200 ms post-stimulus onset (i.e. during the time window corresponding to the MMN) for large deviants only. The same pattern was not reliably seen in the TD group. For the LDN time window (350–550 ms), the DYS group showed a significant difference wave, although this was significant for large deviants only in the younger DYS group and for small deviants only in the older DYS group. The opposite pattern was seen in the TD group (i.e. a significant difference wave occurred during the LDN time window to small deviants for the younger TD group and to large deviants for the older TD group).

#### Mismatch negativity

The size of the MMN response was estimated by taking the mean amplitude of the difference wave over the time window of 100–250 ms [23,48]. The mean amplitudes for the MMN indices for the large and small deviants are shown for the four subgroups in Table 2. Repeated measures ANCOVA showed a main effect of deviant size, *F*(1, 39) = 10.22, *p* = 0.003, with a significantly larger (more negative) MMN to large deviants than to small deviants. There were no main effects of the between-subjects factors DYS status or age band, or of the covariate nonverbal ability, and no significant interactions between factors.

**Table 2:** Mean (SD) MMN and LDN indices for the small and large deviants

		**Group**			
	**Deviant size**	**TD**		**DYS**	
**Age band**		**Younger**	**Older**	**Younger**	**Older**
Deviant mismatch indices					
MMN mean amplitude	Large	-1.77 (10.01)	-3.39 (4.70)	-1.03 (5.36)	-3.33 (7.37)
	Small	1.06 (5.32)	1.76 (9.63)	4.56 (7.08)	3.61 (5.33)
ITC 0–300 ms, 4–7 Hz	Large	0.11 (0.04)	0.08 (0.02)	0.09 (0.02)	0.12 (0.04)
	Small	0.09 (0.02)	0.10 (0.02)	0.12 (0.02)	0.13 (0.02)
LDN mean amplitude	Large	0.41 (11.14)	-2.61 (4.63)	-4.90 (6.12)	-0.92 (5.22)
	Small	-5.47 (5.18)	-0.98 (12.63)	0.62 (7.31)	-3.88 (6.64)
ERSP 300–600 ms, 4–7 Hz	Large	0.04 (0.59)	-0.33 (0.63)	-0.16 (0.39)	-0.11 (0.48)
	Small	-0.02 (0.56)	-0.46 (0.54)	-0.29 (0.52)	0.25 (0.36)

**Figure 4:**
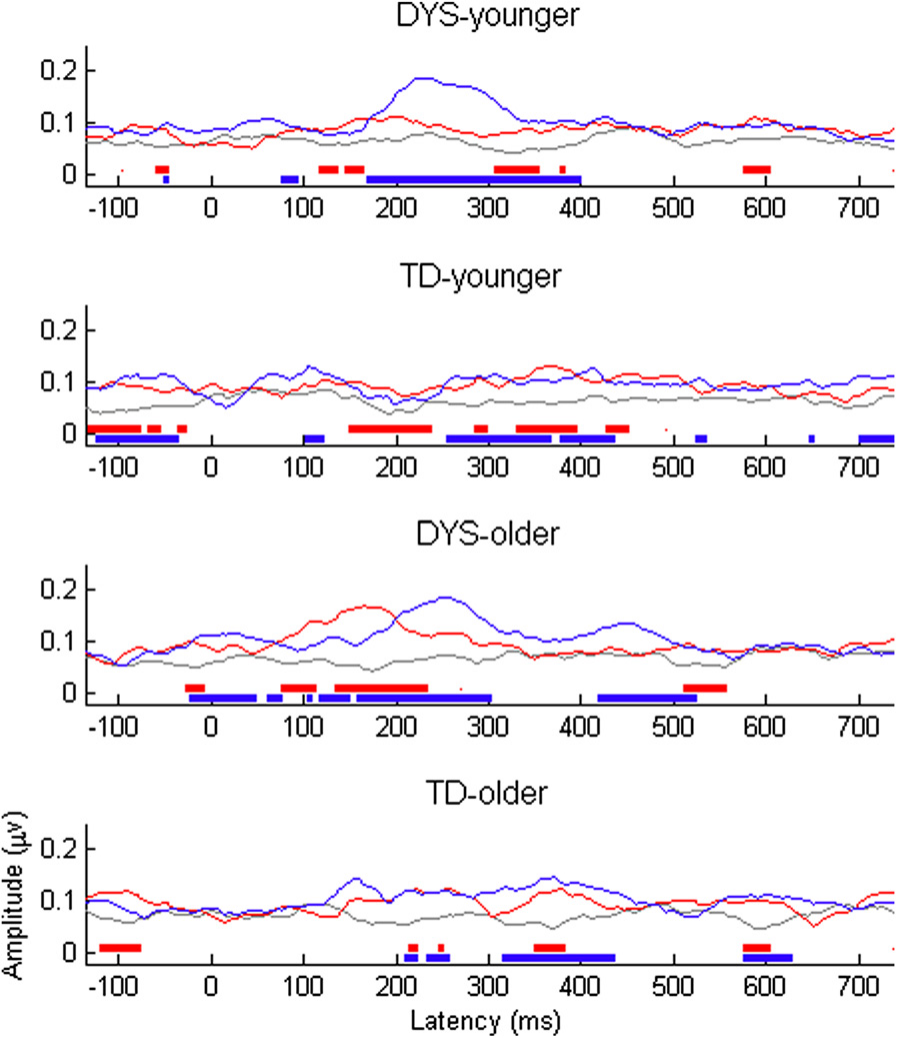
**ITC in the theta range by time for difference waves by DYS status and age band.** Dummy difference files are shown in grey, large deviants in red, and small deviants in blue. Regions where *t* test value is < -1.96 are shown as bars of same colour below the plot.

**Figure 5:**
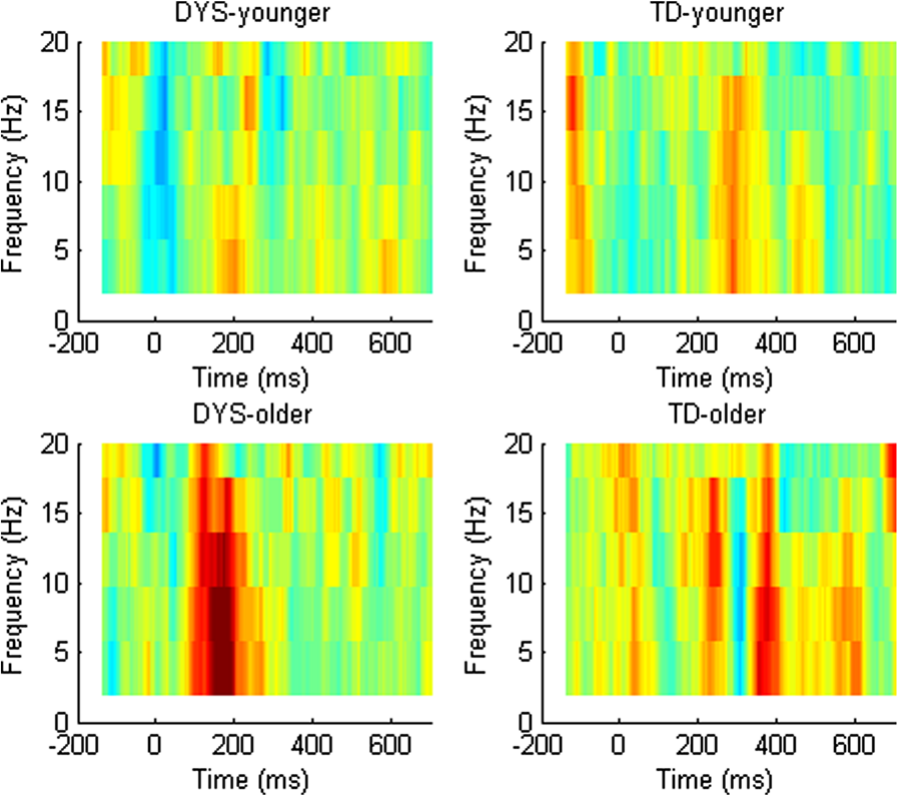
**ITC for difference waves in relation to DYS status and age band.** Colours indicate range from zero (green) to 0.2 (deep red).

Figures 4 and 5 show the mean levels of ITC, along with their spectral components, for mismatch waves in frequencies ranging from 1 to 20 Hz for the four subgroups. As described by Bishop et al. [23], the MMN region is characterized by a burst of increased ITC that is particularly marked in frequencies below 10 Hz. Visual inspection suggested that the DYS groups showed greater synchronization of these frequencies during the MMN time window relative to the controls. To explore this further, we compared the mean ITC in the first half of the trial (0–300 ms) between groups for the small and large deviants (see Table 2). Repeated measures ANCOVA revealed a significant three-way interaction between DYS status, age band, and deviant size, *F*(1, 35) = 4.61, *p* = 0.039. To explore this interaction, we first divided the sample into younger and older age bands. Repeated measures ANCOVA showed that for the older age band, the DYS group showed larger ITCs than the TD group, regardless of deviant size, *F*(1, 18) = 12.93, *p* = 0.002. However, for the younger age band, there was a significant interaction between DYS status and deviant size, *F*(1, 18) = 6.53, *p* = 0.020. Follow-up *t* tests showed that whereas there was no significant difference between the younger DYS and TD groups in the size of their ITC to the large deviant, *t*(1, 18) = 1.34, *p* = 0.198, the younger DYS group had a significantly larger ITC than the younger TD group in response to small deviants, *t*(1, 18) = -3.27, *p* = 0.004.

#### Late discriminative negativity

Following Bishop and colleagues [23,48], the size of the LDN response was calculated as the mean amplitude of the difference wave between 350 and 550 ms post-stimulus onset. Repeated measures ANCOVA showed a significant three-way interaction between DYS status, age band, and deviant size, *F*(1, 35) = 8.10, *p* = 0.007. To explore this interaction further, we divided the participants according to age band and re-ran the model. For the younger age band, there was a significant interaction between deviant and group only, *F*(1, 18) = 10.44, *p* = 0.005. *t* tests showed that the mean LDN to small deviants was significantly smaller in the younger DYS group than in the younger TD group, *t*(1, 18) = -2.15, *p* = 0.045. However, the mean LDN to large deviants was not significantly different between the younger DYS and TD groups, *t*(18) = 1,32, *p* = 0.203. For the older age band, in contrast, there were no main effects of DYS status, or deviant size, and no significant interactions between these.

**Figure 6:**
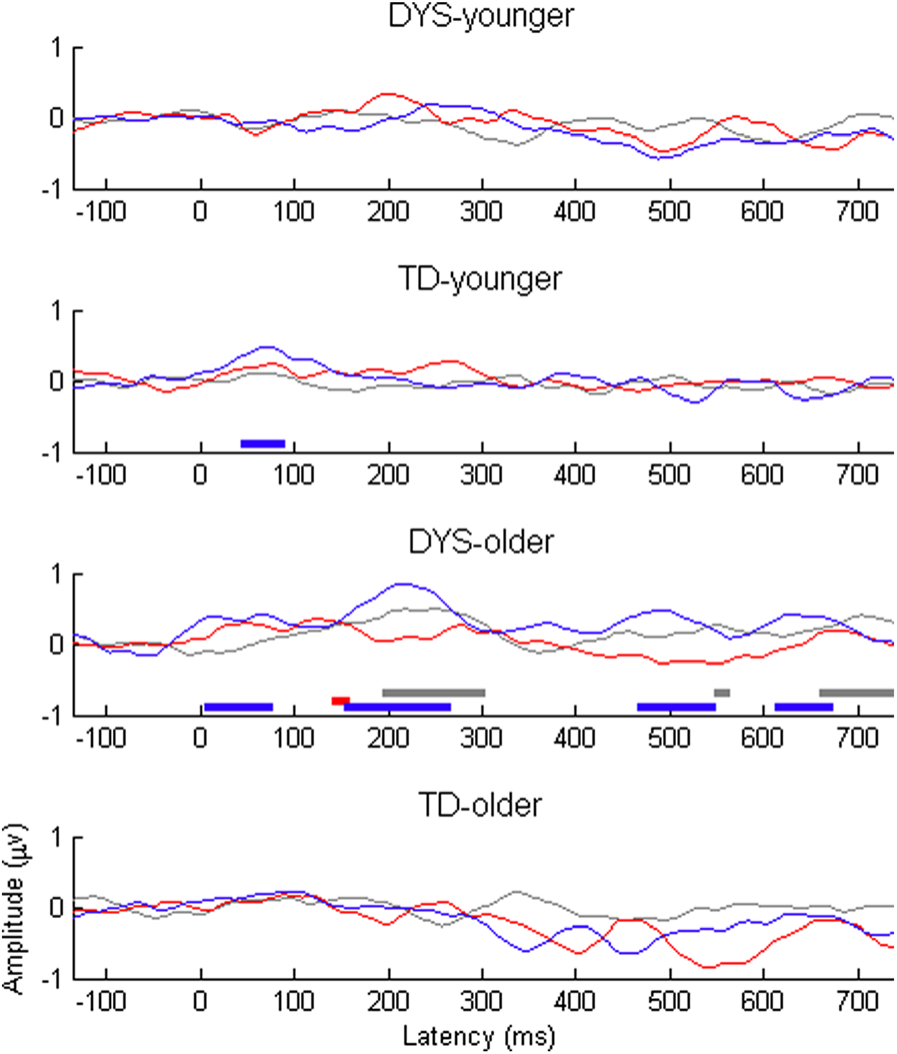
**ERSP in the theta range by time for difference waves by DYS status and age band.** Dummy difference files are shown in grey, large deviants in red, and small deviants in blue. Regions where *t* test value is < -1.96 are shown as bars of same colour below the plot.

**Figure 7:**
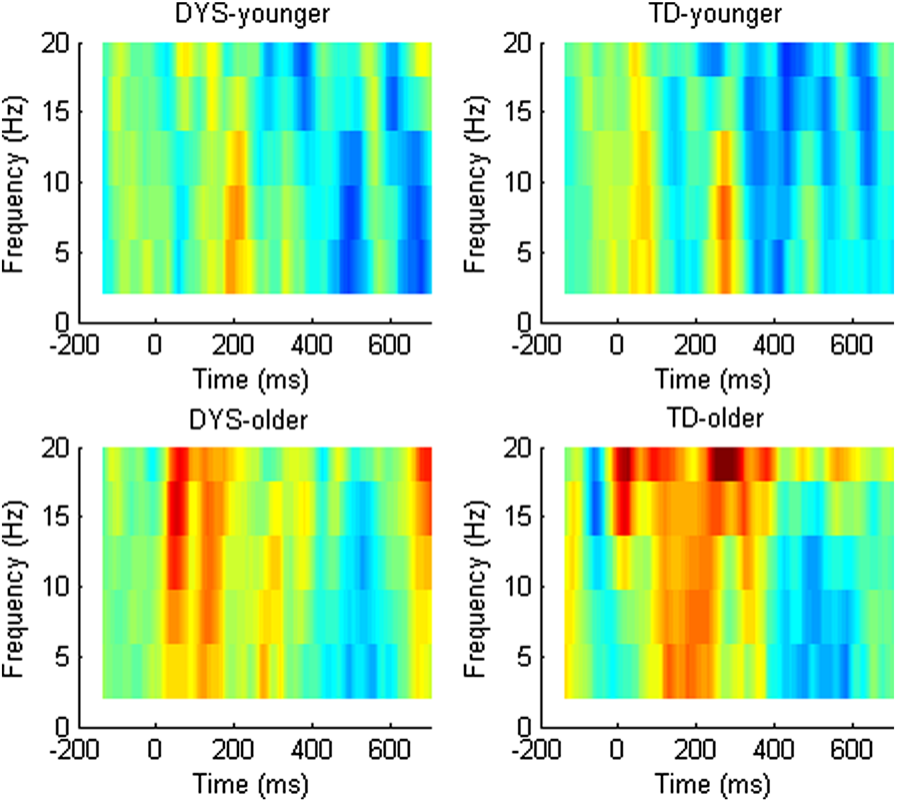
**ERSP for difference waves in relation to DYS status and age band.** Colours indicate range from -0.75 (deep blue) through zero (green) to 0.75 (deep red).

Figures 6 and 7 show corresponding data for the ERSP. As demonstrated by Bishop et al. [48], the plots for the TD group were characterized by a decrease in power across a broad frequency band in the later half of the trial (blue denoting power below baseline levels). The DYS group, in contrast, showed what appeared to be a greater but less prolonged decrease in power that was restricted to frequencies below approximately 13 Hz. Repeated measures ANCOVA was used to compare the mean ERSP in the theta range (4–7 Hz) from 300–600 ms between the DYS and TD groups and between age bands (see Table 2). This demonstrated a significant interaction between group and age band, *F*(1, 35) = 11.55, *p* = 0.0023. We investigated this interaction by dividing the group into younger and older age bands and re-running the model. For the older age band, whereas the TD group showed a large decrease in power over the LDN time window, the DYS group did not, *F*(1, 18) = 8.45, *p* = 0.009. In contrast, the younger DYS group did not differ significantly in their amount of ERSP over the same time window, *F*(1, 18) = 2.49, *p* = 0.132.

### Relationship between indices of mismatch responses and behavioural measures

Finally, we asked whether any of the mismatch indices correlated with any of the questionnaire and psychometric and psychophysical behavioural measures. Following Bishop et al. [23,48], partial correlations (controlling for age) were restricted to the small deviants. For frequency discrimination, separate correlations were run for the DYS and TD groups to account for differences between the stimuli used for the two groups. This resulted in four correlations per group and a Bonferroni adjusted *p* value of 0.013 (see Table 3). We did not replicate the finding by Bishop et al. [48], of an association between frequency discrimination and early ITC, or between frequency discrimination and any of the measures of the MMN or LDN, for either of the groups.

**Table 3:** Pearson partial correlation coefficients (±95% confidence intervals) between frequency discrimination thresholds and MMN and LDN indices

**Small deviant mismatch indices**	**TD group**	**DYS group**
MMN mean amplitude	-0.17 (-0.71–0.30)	-0.03 (-0.59–4.1)
ITC 0–300 ms, 4–7 Hz	-0.42 (-0.86–0.03)	0.03 (-0.64–0.54)
LDN mean amplitude	0.04 (-0.48–0.46)	-0.10 (-0.57–0.28)
ERSP 300–600 ms, 4–7 Hz	-0.22 (-0.68–0.27)	0.08 (-0.52–0.56)

Correlations between the mismatch indices and the questionnaire and psychometric measures are shown in Table 4. In order to control for possible effects of age, the mismatch indices were standardized residuals based on age of the control group. Significance levels were Bonferroni adjusted to *p* = 0.003, controlling for 20 comparisons. Significant correlations are displayed in Additional file 2: Figure S8. After controlling for age, there was a significant correlation between MMN amplitude and GCC scores on the CCC-2, with increasingly negative MMN amplitudes associated with higher GCC scores (i.e. better language skills). There were also significant negative correlations between early ITC and the GCC, TOWRE phonemic decoding, and NEPSY nonword repetition tests, driven by the higher ITC scores of the DYS groups and their correspondingly poorer scores on the psychometric tasks. Finally, there was a significant correlation between ERSP and scores on the Matrices Reasoning subtest from the WASI, with increasing desynchronization associated with higher nonverbal ability scores. Only the correlation between early ITC and nonword repetition remained significant after controlling for multiple comparisons.

**Table 4:** Pearson correlation coefficients between questionnaire and psychometric measures and MMN and LDN indices

**Small deviant mismatch indices**	**WASI matrices**	**TOWRE sight words**	**TOWRE phonemic decoding**	**NEPSY nonword repetition**	**CCC-2 GCC**
MMN mean amplitude	0.10 (-0.23–0.39)	-0.14 (-0.48–0.16)	-0.06 (-0.49–0.31)	–0.25 (-0.58–0.11)	–0.35 (-0.59 to -0.07)*
ITC 0–300 ms, 4–7 Hz	–0.21 (-0.48–0.06)	-0.27 (-0.50 to -0.03)	-0.36 (-0.60 to -0.11)*	-0.53 (-0.72 to -0.30)**	-0.37 (-0.63 to -0.05)*
LDN mean amplitude	-0.01 (-0.24–0.23)	0.02 (-0.36–0.36)	0.12 (-0.31–0.49)	-0.19 (-0.48–0.13)	-0.12 (-0.37–0.16)
ERSP 300–600 ms, 4–7 Hz	-0.36 (-0.60 to -0.03)*	0.02 (-0.26–0.30)	-0.15 (-0.41–0.13)	-0.11 (-0.38–0.20)	-0.13 (-0.42–0.18)

Correlations reported across groups (*N* = 40). Parentheses indicate ±95% confidence intervals. *p = 0.05 (significant); **p = 0.001 (significant).

## Discussion

The current study asked: Do children with dyslexia show differences in their mismatch responses to frequency deviants during the MMN time window using (1) conventional amplitude measures and (2) time-frequency analysis?, and (3) is performance on any of these measures linked to behavioural measures of frequency discrimination, language, and literacy? We found that children with dyslexia did not differ from controls in their MMN responses to the frequency deviants and, surprisingly, that they showed *greater* levels of ITC (phase locking) in the time window corresponding to the MMN, particularly in the older group. In contrast, for the LDN time window, the younger children with dyslexia showed a smaller LDN response to small deviants, whilst the older children with dyslexia showed a reduction in their event-related desynchronization to frequency deviants over the same timescale, in the form of a less negative ERSP. Neither the conventional nor time-frequency analyses scores correlated with performance on a behavioural frequency discrimination task. However, higher ITC scores for the MMN were associated with *poorer* performance on a test of nonword repetition.

Our failure to find a difference between the dyslexic and control groups on the average amplitude measure of the MMN adds to a growing number of studies that have reported similar results [28-32]. However, as outlined earlier, the literature is by no means consistent as several studies have reported evidence for a reduced MMN to tone deviants in adults with a history of dyslexia (both compensated and noncompensated [25,26]), whilst Lachmann and colleagues [37] reported the same in a group of children with dyslexia, but only in those who showed difficulties with word but not nonword reading (c.f. [24]). In contrast, Hugdahl et al. [27] found that the MMN was enhanced in a group of 25 children with dyslexia. Bishop [40] undertook an extensive review of the literature to better understand the reasons for inconsistencies in findings in the literature. She identified a number of relevant factors including differences in statistical power, stimuli, time windows, and presentation rates (see also [39] for a review). Our findings reinforce Bishop's [40] conclusions. First, regarding power, with a sample size of 20 in each group and 20 in each age band, our study was powered at 0.69 to detect a strong effect size, so it is possible a small effect could have been missed. However, the fact that we saw a trend for the dyslexic children to show a *larger* MMN than controls suggests that if such an effect did exist in our data, it was likely to be in the opposite direction than would be expected were deficits to exist at this stage of processing. Second, our findings support an increasing consensus that when significant group differences are detected, they are likely to arise from studies where the frequency difference between standards and deviants is small (<10%; [39,40]). Although we did not find an effect of deviant size on the MMN, the younger dyslexic group showed a reduction in their LDN response to small deviants only. This brings us on to the question of time windows. To the extent that our study provided evidence for differences in late-stage auditory processing in dyslexia (as measured by the LDN and associated ERSP), it may be tempting to conclude that those studies reporting a difference between dyslexic and control samples may have been conflating the MMN and LDN time windows (see [48] for similar argument). However, close examination of the time windows reported in the above cited studies yields no evidence that this is the case. Nevertheless, that we were interested in the time window associated with the LDN may have contributed indirectly to our null results. Indeed, Bishop [40] noted a strong trend for significant differences in the MMN to tone deviants to arise from those studies using a stimulus onset asynchrony of 500 ms or less. Because we were also interested in late-stage processing, we did not do this. However, it is possible that differences in the MMN to tone deviants between dyslexic and control groups only arise when the frequency difference between standard and deviant stimuli is small *and* the presentation rate is fast.

Together with the MMN result, our finding that the dyslexic group showed an enhanced ITC to frequency deviants relative to controls suggests that children with dyslexia have no difficulty with the initial detection or discrimination of sound differences, nor in the precision of synchronization of cortical oscillatory activity corresponding to these abilities. This deduction is of particular interest in light of two recent theories which have attributed the reading difficulties associated with dyslexia to difficulties in cortical phase locking [2,3]. Specifically, Goswami [3] argued that dyslexia arises from a reduction in oscillatory phase locking in auditory cortex to slower temporal modulations, in particular to delta and theta ranges (0.5–4 and 4–8 Hz, respectively). Giraud and Ramus [2] instead implicated a deficient low-gamma steady state response in the left auditory cortex. We could not ascertain whether the children in our sample had deficits in the phase locking of their gamma response, owing to the application of an online notch filter in our study. However, in as much as we showed evidence for enhanced phase locking at 4–7 Hz, our study indicates that children with dyslexia are unlikely to have a deficit in generating phase-locked oscillations in the theta range in response to steady-state (nonmodulating) auditory stimuli. It is also worth noting that our findings are not incompatible with those of two recent MEG studies, both of which examined phase-locking activity to non-steady-state (modulating) noise in two separate groups of adults with a childhood history of dyslexia [70,71]. Lehongre et al. [71] found that their dyslexic group showed a reduction in cortical phase locking in the left hemisphere to acoustic modulations in the low gamma range (25–35 Hz). Poorer phase locking over this frequency range was linked to greater deficits in phonological processing and rapid naming. In contrast, Lehongre et al. [71] also found evidence for enhanced cortical entrainment to high gamma rates (>50 Hz) in their dyslexic sample, which was linked to a verbal memory deficit. Hämäläinen et al. [70] reported evidence for a differential pattern of phase locking at 2 Hz in the cortices of their dyslexic group; phase locking was reduced in the right hemispheres of their dyslexic subjects relative to those of controls, and the dyslexic group showed a more bilateral distribution of responses than normal readers. However, Hämäläinen et al. [70] failed to find any group differences at 4 Hz and, moreover, also reported evidence for enhanced phase locking in the left hemispheres of their dyslexic group at 10 and 20 Hz. Together with the findings of Hämäläinen et al. [70] and Lehongre and colleagues [71], our results suggest that if cortical phase locking is impaired in individuals with dyslexia or with a history of the disorder, this impairment is unlikely to be in the theta range.

The results of our study also shed further light upon what stage(s) of auditory processing is (are) likely to be impaired in dyslexia. Our analyses of the LDN and corresponding ERSP indicate that the difficulties children with dyslexia experience in processing deviations in auditory information may arise at a relatively late stage of processing (i.e. following initial detection and discrimination). However, our findings were not clear cut. Indeed, whereas the younger children with dyslexia showed a reduction in their LDN response to small deviants, the older children with dyslexia did not differ from controls in their LDN amplitude to either small or large deviants. Instead, this same group showed a reduced ERSP relative to the controls during the same time window, which was absent in the younger children with dyslexia. Clearly, these findings need to be replicated, and preferably over a wider age range than that studied here, in order to understand these developmental effects. However, the fact that we observed a reduction of broad, low frequency event-related desynchronization over this time window in the older children with dyslexia suggests that this group may maintain subtle differences in their late-stage auditory processing that can no longer be detected in the difference wave.

Inasmuch as our findings suggest that the auditory deficit in dyslexia occurs in late—but not early—stage processing, they are consistent with other recent findings from the literature [72]. Neuhoff et al. [72] measured the MMN and LDN to speech signals in children with dyslexia, their unaffected siblings, and controls [72]. They found that whilst the MMN was not significantly different between groups, both the children with dyslexia and their unaffected siblings showed an LDN that was significantly reduced relative to that of controls, a finding that was used to argue that the LDN may represent a neurophysiological endophenotype for dyslexia. Our findings are also consistent with those of Bishop et al. [48], who showed that children with SLI showed a reduction in their LDN response to small deviants and failed to show the event-related desynchronization during the LDN time window that was characteristic of age- and nonverbal IQ-matched controls. However, how far our findings indicate that this pattern may extend to children with reading but not oral language difficulties remains uncertain. Indeed, the children with dyslexia in our study had poorer scores than the controls on both the parental report questionnaire of communication (CCC-2) and on a test of nonword repetition, suggesting that at least some of them had oral language problems in addition to poor reading. This is hardly surprising, as many children with dyslexia also meet criteria for SLI, and vice versa [51,73,74], and indeed several theories purport a shared causal mechanism underlying both disorders (e.g. [3,5,50]). Nonetheless, our findings suggest that the pattern of reduced desynchronization of neural activity during late-stage processing at least extends to (older) children who have difficulties with learning to read in addition to oral language problems and that this may be a marker for developmental disorders of oral and written language.

Finally, our results add to an increasingly complicated picture regarding the relationship between auditory processing abilities (measured both behaviourally and electrophysiologically) and language and literacy outcomes in children. First, we failed to replicate the findings of Bishop et al. [23] for a positive correlation between ITC to small deviants and performance on psychophysical measures of frequency discrimination, in either the dyslexic or the control groups. Nevertheless, because the analyses were within-group, our sample sizes were small. However, it is noteworthy that Bishop et al. [48] equally did not find evidence for correlations between frequency discrimination thresholds and any of the electrophysiological measures they obtained in groups of typically developing children and children with SLI. We also failed to replicate the finding of Bishop et al. [48] for a significant correlation between nonword repetition and event-related desynchronization. Rather, the only correlation that remained significant in our study after controlling for multiple comparisons was between precision of phase locking during the MMN time window and nonword repetition, although this was driven by the combination of both *higher* ITC scores and *lower* nonword repetition scores in the dyslexia group.

In so far as auditory processing as measured in this study was not strongly associated with behavioural measures of oral and written language, our results question the causal link that has been purported to exist between auditory processing deficits, in this case frequency discrimination, and developmental disorders of language and literacy (e.g. [2-5]). At the same time, it is increasingly clear that both children and adults with dyslexia (and indeed many with SLI) do experience difficulties with auditory processing, as measured using both behavioural and electrophysiological, as well as imaging techniques (for reviews, see [9,40]). We have previously argued that the link between auditory processing deficits and developmental disorders of language may be noncausal, perhaps driven by the existence of a third factor [75]. However, another possibility that has been put forward is that rather than being a cause of reading/language impairments, deficits in auditory processing might instead be a consequence [76]. Using structural equation modeling, Bishop and colleagues [76] found that the relationship between auditory processing, family history, and phonological processing in SLI was best predicted by a model where family history fed into phonological processing, which then impacted upon auditory processing. According to this model, genetic risk factors may affect a child's ability to develop phonological categories, leading to changes in the way that sound is represented in the brain, as measured by the ERP. This would predict that auditory processing deficits and difficulties with language and literacy should co-exist (as they indeed do). However, it would not require poorer auditory processing to be associated with more severe behavioural symptoms, as a number of different genetic risk factors could lead to different profiles of behavioural strengths and weaknesses (see also [74]). Clearly, further research is needed to support this hypothesis. Large-scale longitudinal studies with *a priori* predictions would go a long way towards achieving this.

## Conclusion

The current study demonstrates that children with dyslexia may show unimpaired cortical processing regarding the initial detection and/or discrimination of sound differences in the frequency domain. Rather, such auditory processing deficits may instead manifest in dyslexia at a relatively late stage of processing. These findings are consistent with the notion that a reduction in the LDN, or the broad, low-frequency event-related desynchronization associated with the LDN, may represent a neurophysiological endophenotype of dyslexia.

## Competing interests

The authors declare that they have no competing interests.

## Authors' contributions

LH gathered data with dyslexic children, analysed the data, and wrote the manuscript. DB designed the original study on which this one was based and assisted with data analysis and interpretation and manuscript revision. MH created the stimulus materials and programmed their presentation. MH and JB recruited and collected data from the control sample. All authors read and approved the final manuscript.

## Supplementary Material

Additional file 1: Table S5 Pearson correlation coefficients between the number of artefact-free epochs and MMN and LDN indices. Click here for file

Additional file 2: Figure S8 Significant correlations between the mismatch indices (standardised for age) and the questionnaire and psychometric measures. Click here for file
