# Supplementary material for: Late, not early mismatch responses to changes in frequency are reduced or deviant in children with dyslexia: an event-related potential study
Source: J Neurodev Disord. 2014 Jul 25;6(1):21. doi: 10.1186/1866-1955-6-21 (PMC4126817; doi:10.1186/1866-1955-6-21)
Supplement: Additional file 1: Table S5 — Pearson correlation coefficients between the number of artefact-free epochs and MMN and LDN indices. [file 1866-1955-6-21-S1.docx]

**Table S5** Pearson correlation coefficients between the number of artefact-free epochs and MMN and LDN indices.

| Deviant size | Large | Small |
| --- | --- | --- |
| MMN mean amplitude | .28 | -.06 |
| ITC 0-300 ms, 4-7 Hz | -.19 | -.12 |
| LDN mean amplitude | -.10 | .17 |
| ERSP 300-600 ms, 4-7 Hz | .11 | -.04 |

* significant at *p* = .05
