# Supplementary figures and images for: Late, not early mismatch responses to changes in frequency are reduced or deviant in children with dyslexia: an event-related potential study
Source: J Neurodev Disord. 2014 Jul 25;6(1):21. doi: 10.1186/1866-1955-6-21 (PMC4126817; doi:10.1186/1866-1955-6-21)

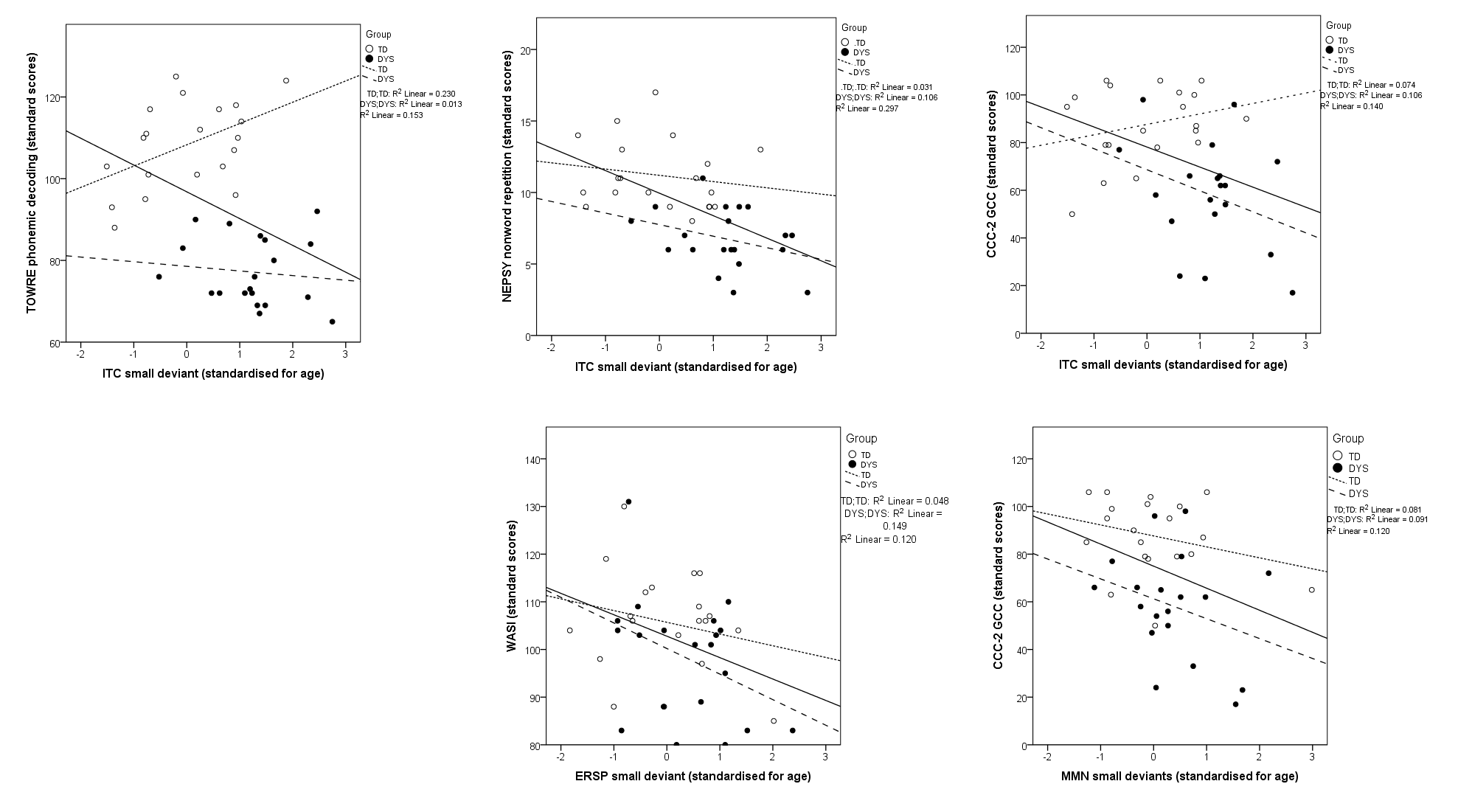

Supplement: Additional file 2: Figure S8 — Significant correlations between the mismatch indices (standardised for age) and the questionnaire and psychometric measures. [file 1866-1955-6-21-S2.tiff]
